# Enhancing Corrosion Resistance and Hardness Properties of Carbon Steel through Modification of Microstructure

**DOI:** 10.3390/ma11122404

**Published:** 2018-11-28

**Authors:** Wilson Handoko, Farshid Pahlevani, Veena Sahajwalla

**Affiliations:** Centre for Sustainable Materials Research and Technology (SMaRT Centre), School of Materials Science and Engineering, University of New South Wales (UNSW Sydney), Sydney, NSW 2052, Australia; f.pahlevani@unsw.edu.au (F.P.); veena@unsw.edu.au (V.S.)

**Keywords:** corrosion resistance, hardness properties, multi-phase microstructure, carbon steel, in-situ confocal microscopy, construction, microstructural engineering

## Abstract

Steel has played a primary role as structural and fabricating materials in various industrial applications—including the construction sector. One of the most important properties of steel that required a constant improvement is corrosion resistance specifically in corrosive environment. For this purpose, various approaches have been conducted through different heat treatment parameters to compare its microstructural engineering on chemical and mechanical properties. In this paper, correlation of different microstructure on corrosion resistance and hardness properties have been investigated. Three different heat treatment cycle have been applied on carbon steel with same composition to prepare dual-structure (DS) steel that consisted of ferrite/pearlite and triple-structure (TS) with ferrite/pearlite/bainite and ferrite/bainite/martensite. Phase transformation during heat treatment process was analyzed through in-situ ultra-high temperature confocal microscopy. Effect of corrosion behavior on these steels was investigated by Tafel plot, Scanning Electron Microscopy (SEM), 3D laser scanning confocal microscopy (3DLSCM), and calculation of phase volume fraction by ImageJ. Mechanical test was conducted by Vickers hardness test. It has been found that TS steels that have improvement in corrosion resistance accounted around 5.31% and hardness value for up to 27.34% more than DS steel, because of tertiary phase—bainite/martensite. This corrosion rate was reduced due to decreased numbers of pit growth and lower level of boundary corrosion as bainite/martensite phases emerged.

## 1. Introduction

In recent decades, microstructural engineering of different carbon steels has received enormous attention, due to its superior properties—strength, hardness, high-cycle fatigue that can be achieved without additional cost to the input materials and through heat treatment process, which is highly used in automotive, gas, oil, and construction industries [1]. One of the main essential properties of steels in which often demanded a constant improvement is its corrosion resistance. To understand the effect of microstructural engineering on the corrosion behavior, an investigation of each phase is crucial to determine the combination of phases that added a positive impact on corrosion resistance properties in steel. This study is based on the extended research on influence of different microstructural phases on corrosion behavior in high carbon steel [2] and has been employed on low carbon steel. In low carbon steel, the DS steel is commonly characterized by a group of ferrite (F) matrixes and martensite (M) phases [3,4] or ferrite and bainite (B) phases [5]. With the increasing utilization of more corrosive solutions in various industrial applications to minimize the manufacturing cost, it has escalated the influence of corrosion in the failure of steel parts. Nonetheless, the effect of aging on those applications, quantification of all aspects, which will assist to the long-term failure has become more crucial. One of the major aspects is the chloride effect, such as the de-icing process, which is commonly utilized in winter season, and various structural applications are used in marine environment that will lead to corrosion. The corrosion defects not only concern on the appearance of its external structure, but also the safety with subsequent decrease strength properties through internal and cross section regions of steel.

From the microstructural point of view, previous research proposed that corrosion rates can be minimized by balancing DS steel—ferrite (F) and pearlite (P) microstructures, since the second phase promoted the negative side-effect on the corrosion resistance in which lead to dissimilarity in compositional and matrix-lattice structure that corresponded to different free energy of each individual phase [2,6]. Additionally, in long term immersion in corrosion solution, this DS steel had advantages over the steel with more homogeneity of its microstructure, such as single-structure (SS) mild steel [7,8]. This occurrence can be described from thermodynamic perspective, due to the free energy of SS (e.g., martensitic phase), which was higher than ferrite and pearlite; hence, Fe atoms were more susceptible towards corrosion reaction with ions dissolved in media [9].

A recent study by Handoko et al. [10,11] stated that preferential corrosion attack on retained austenite was then followed by martensite in DS steel, due to the martensite islands having possessed higher-levels of carbon content. Another factor that caused this phenomenon was the effect of small size, shape, and volume fraction of retained austenite phase that caused more boundary-to-boundary contact between its grain boundary interfaces and martensite plates that led to higher grain boundary corrosion [11]. A very limited investigation on triple-phase (TS) steel, according to Park, et al. [12], alleged that ferrite/bainite/austenite steel performed at lower corrosion rates than DS steel, due to the most inferior corrosion resistance in TS steel, which was bainite with volume fraction of 40%, whereas in DS steel, which was martensite with only 16%. Moreover, the presence of the third phase not only improved the corrosion resistance, but also its hardness properties; thus, enhancement on structural integrity not only enhanced the mechanical properties, but also reduced its dependency on additional alloying elements. Although those dual- and triple- phases had shown different corrosion behaviors, this specific grade of steel with ferrite, bainite, martensite combination has not been studied yet.

In-situ observation by ultra-high temperature laser microscope provided high resolution images to observe microstructural changes through heat treatment process and different quenching rate. At room temperature, the final phases were observed by an optical polarized microscope. A powerful technique of Tafel polarization analysis was used in this research to measure the effect of this tertiary phase on corrosion current rate. This electrochemical test was capable to offer valuable quantitative and chemical data for corrosion study [13], but it was not able to observe the surface evolution during the corrosion test. Scanning Electron Microscope (SEM) provided high resolution morphological imaging employing focused beam of electrons on surface of steel sample, but it is not able to undertake surface roughness measurement. 3D Laser Scanning Confocal Microscope (3DLCSM) offered non-destructive analysis with high reconstruction morphological imaging to measure segmented surface roughness changes in nano-scale depth of each different phase by corrosion. Greyscale images converted after reconstruction were evaluated to calculate volume fraction of DS and TS steels by using image analyzer software, ImageJ [14].

After taking into consideration the complexity of corrosion mechanism attributed to combination of different phases, its corrosion behavior should be defined by incorporating theoretical and experimental analysis. The research concerning corrosion resistance of different phase combinations on this grade of TS steel has not been studied and still scarce in literature of investigation on DS steels in details [15,16], as most studies were focused on microstructural corrosion behavior on SS steels [17]. The aim of this investigation is to demonstrate and elucidate the effect of different microstructural engineering on low carbon steels with the same composition that will lead to combination of phases for improving corrosion resistance and hardness properties, eliminating costly and time-consuming heat treatment and dependency on alloying elements.

## 2. Materials and Methods

### 2.1. Material Preparation

**Figure 1:**
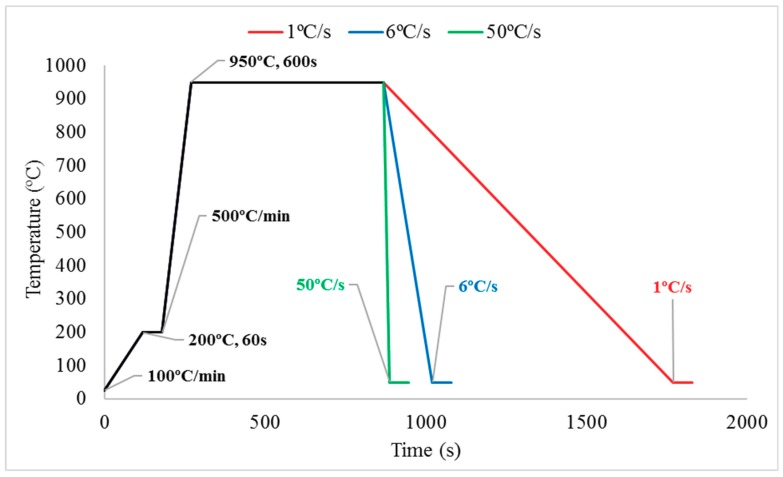
The schematic diagram of different quenching rate experiments for formation of DS and TS steels.

Carbon steels with 0.20 C, 0.67 Si, 0.61 Mn, 0.05 Cr, and 0.10 Ni (in wt. % unit) have been utilized in this investigation. The steels were slowly cut to dimension of 10 × 10 × 4 mm at 100 rpm feeding rate by Struers Minitom (Struers, Rødovre, Denmark) that were equipped with a diamond blade to prevent heat transfer and stress that can transform its existing phases. Three cut samples were grinded with SiC-based paper to 4000 grits by Struers TegraPol-21 (Struers, Rødovre, Denmark) and mirror-polished to 1 μm by Struers Rotopol-22 (Struers, Rødovre, Denmark) to fit into 10 mm-diameter alumina crucibles for heating and quenching processes by in-situ observation on confocal microscope CLM VL2000DX (Lasertec, Yokohama, Japan). At the initial stage, the chamber of confocal microscope was vacuumed to eliminate gases that will lead to oxidation process during the test. 99.99% pure Ar gas was pumped into chamber for three to four times for about 2.5 min per cycle. The He gas valve was kept in ready mode for the quenching process. A sample was then heated to 200 °C for 60 s to avoid pressure and thermal shock from its outside chamber, then heated to 950 °C with holding time of 600 s and a different quenching rate was applied at 1 °C/s, 6 °C/s and 50 °C/s, as shown in Figure 1. Determination of mean intercept austenitic grain size can be conducted through a diagonal line overlaid from the top left to bottom right section based on the standard procedure from ASTM grain size number conversion. Nine section images of each steel before quenching were captured and calculated. The 3.5 wt. % of NaCl solution was prepared and used for corrosion experiment for up to two hours at room temperature, 24 °C, to simulate the chloride-effect-like condition. All steel specimens were ultrasonically cleaned by ultrasonic cleaner (Unisonics Australia, Brookvale, Australia) to remove remaining corrosion product and dirt, then finally dried by Struers Drybox-2 (Struers, Rødovre, Denmark).

### 2.2. Analytical Methods

Microstructural investigation was carried out by using optical microscopy Nikon Eclipse ME-600 (Nikon Corporation, Tokyo, Japan) after light etched by 2% Nital solution to prevent unnecessary corrosion degradation process. Two corrosion test experiments had been conducted in this research, electrochemical measurement and immersion technique. Electrochemical corrosion test was conducted by Versatile Multipotentiostat VSP300 (EC-Lab^®^, Claix, France) and acquired quantitative data was compiled by EC-Lab v11.02 software. This instrument was linked to the corrosion cell kit with three different cables to electrodes (saturated calomel electrode (SCE) as a reference electrode, platinum foil as counter electrode, and low carbon steel sample as working electrode). 3.5 wt. % NaCl solution was prepared as electrolyte and the tested steel sample area was 1 cm^2^ in open circuit potential (OCP) system with immersion time of 2 h. The obtained Tafel polarization curves were compiled at potential between (±250 mV vs. OCP) to (±250 mV vs. OCP) and scan speed at 0.5 mV/s. Volume fraction of each phase was precisely measured by image analyzer software, ImageJ 64-bit Java 1.8.0.112 version. With four different parts of imaging were captured for each sample, to obtain average volume fraction value of a single phase. Scanning Electron Microscope (SEM)—Hitachi S3400I (Hitachi High Technologies America Inc., Schaumburg, IL, USA) was used to observe the morphological surface changes by immersion technique. A high-resolution, three-dimensional imaging from 3DLSCM VK-X250 (Keyence International, Mechelen, Belgium) was used to analyze microstructural corrosion before (after light etched) and after corrosion test to define which phase was more or less susceptible to preferential attack by immersion technique. Overall corrosion behavior of each phase was compared and summarized through evolution of its mean surface roughness depth (Rz) in nano-scale unit by MultiFileAnalyzer software. Line scan profile of approximately 22 μm-line roughness on each microstructural interface was plotted from data critical analysis. A mechanical measurement for hardness properties of produced microstructures on steel was determined by Struers Hardness Tester DuraScan-20 (Struers, Darmstadt, Germany), ASTM E140.

## 3. Results and Discussions

**Figure 2:**
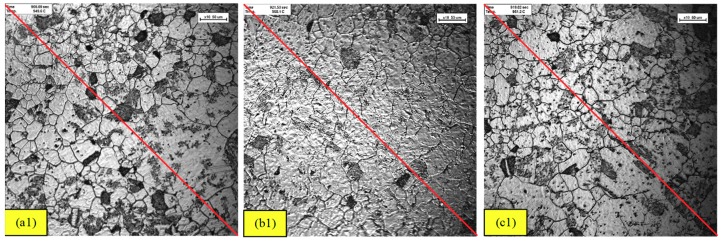
In-situ observation by confocal microscopy at the last seconds before quenching process. Temperature at around 950 °C before different quenching rate (**a1**) 1 °C/s, (**b1**) 6 °C/s and (**c1**) 50 °C/s.

Prior to the corrosion test, the investigation on microstructural changes through effect of different quenching rate was imperative to understand the formation of various phases. In-situ observation from ultra-high temperature laser microscope were recorded and analyzed a second before a different quenching rate was applied to the heated steels, as shown in Figure 2. The Figure 2(a1)–(c1) indicated the steel at temperature of 950 °C on holding time of 600 s before quenching was applied to 1 °C/s, 6 °C/s, and 50 °C/s rates, respectively. From these figures, the ASTM average grain size number accounted to 5.5. This value was converted to mean intercept grain size of 47.6 ± 0.5 μm for all samples, thus, the effect of parameter can be eliminated. Therefore, with different phase created after heat treatment, it will lead to distinct corrosion behavior from each combined phase.

**Figure 3:**
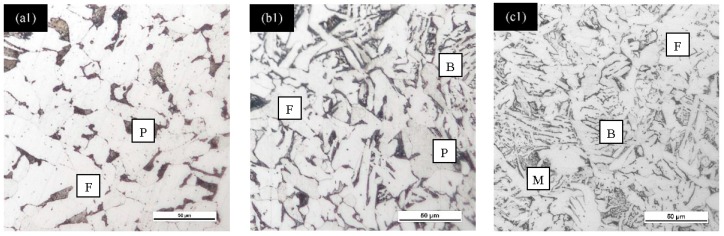
Optical imaging of different quenching rate at (**a1**) 1 **°**C/s, (**b1**) 6 **°**C/s and (**c1**) 50 **°**C/s for carbon steel samples after light etching. Where F: Ferrite, P: Pearlite, B: Bainite, M: Martensite.

The macroscopic morphological optical imaging analysis was undertaken to observe various microstructure formations as the influence of different quenching rate on carbon steel samples. The slowest quenching rate at 1 °C/s produced DS of ferrite/pearlite, whereas cooling rate at 6 °C/s and 50 °C/s generated TS of ferrite/pearlite/bainite and ferrite/bainite/martensite, respectively, as per the continuous-cooling-transformation (CCT) diagram [18], as shown in Figure 3. It can be observed that F has a bright color phase, P possesses dark lamellar or layered microstructure, B refers to dark wedge-shaped plate, and M is classified as dark compacted lath-shaped structure. This microstructural dissimilarity will lead to different corrosion behavior on each steel. Powerful electrochemical measurements were carried out to define the corrosion rate of each sample in details to provide valuable quantitative data to summaries overall corrosion behavior.

**Figure 4:**
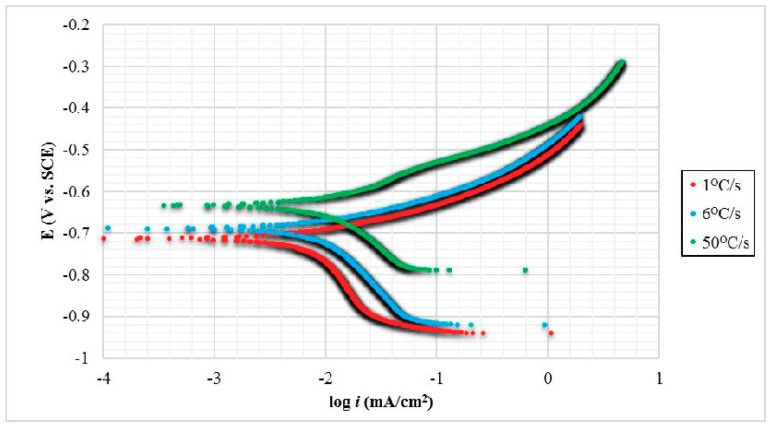
Tafel extrapolation diagram of electrochemical corrosion measurements of different quenching rate on low carbon steel samples. E_corr_: Corrosion potential, i_corr_: Corrosion current rate.

Electrochemical corrosion tests were carried out to measure the corrosion resistance properties of carbon steels, as various phases were formed by effect of different quenching rate. This corrosion measurement technique was used to define each of the Tafel polarization curves and corrosion current rates, i_corr_, through the cross-line between anode line (upper section—discharging), which refers to metal dissolution reaction, and cathode line (lower section—charging), which classifies as transformation reaction of H_2_, horizontally passed through corrosion potential value, E_corr_. Figure 4 represents experimental polarization curves for three carbon steel samples with different quenching rate after immersion in 3.5 wt. % NaCl electrolyte for 2 h. With lower i_corr_ and more positive E_corr_ values, it performs improvement on corrosion resistance properties in steel. The logarithm of i_corr_ values were recorded with the least corrosion rate value on TS sample-50 °C/s. On the contrary, the highest corrosion rate was attributed to sample-1 °C/s, followed by sample-6 °C/s. TS sample-50 °C/s had lower corrosion potential, E_corr_, displaced of approximately 9.16% and 12.48% more than TS sample-6 °C/s and DS sample-1 °C/s, respectively, toward a more noble side. These discrepancies can be corresponded to different corrosion behavior of each microstructural phase.

Data were collected and calculated to obtain E_corr_ and i_corr_ values of each steel sample as shown in Table 1. It presented that different carbon steel properties as the influence of different quenching rate that correlated to distinct corrosion potential value in which shifted to a more negative direction had less corrosion resistance properties. This means that the sample with the highest quenching rate was the most stable at low negative value, exhibiting that fast cooling reduced its propensity on corrosion attack. It was found that the DS—ferrite/pearlite had the non-preferable electrochemical corrosion properties, followed by TS steels of ferrite/pearlite/bainite and ferrite/bainite/martensite phases, respectively. This is due to the fact that ferritic phase possessed the lowest C contents than other phases. Additionally, ferrite acted as anode due to more Fe content, whereas pearlite acted as cathode, since pearlitic phase reverted to C enrich austenitic phase [19]. Another aspect that led to TS steel had better corrosion resistance was because of lower ferritic volume fraction and an increase of pearlite/bainitic, bainitic/martensitic phase that had lower Fe content and packed crystalline structure. Each microstructural property investigation in detail towards corrosion attack and determination of volume fraction will be further proven by SEM, 3DLSCM, and ImageJ.

**Figure 5:**
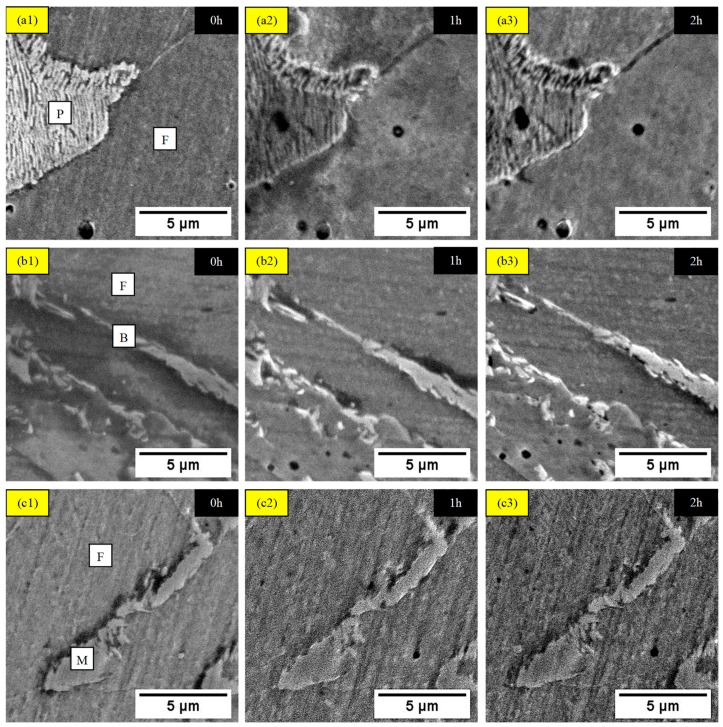
SEM imaging of surface evolution by different time of corrosion for (**a1**), (**a2**) and (**a3**) DS ferrite/pearlite, (**b1**), (**b2**) and (**b3**) TS ferrite/bainite, (**c1**), (**c2**) and (**c3**) TS ferrite/martensite in 3.5 wt. % NaCl solution.

High-resolution morphological images were analyzed by SEM analysis with Secondary Electron (SE) mode to provide evolution of phase changes over 2 h corrosion test, as presented in Figure 5. Although all morphological images were finely polished and lightly etched, the surface of steels were roughed because of phase differentiation in corrosive solution. It can be observed that at t = 0 h, the different pair of phases were revealed, and each microstructural phase was clearly distinguishable. The pair of phases consisted of DS ferrite/pearlite that acted in the similar way for all corrosion behavior in TS ferrite/pearlite in both TS steels—TS ferrite/bainite corrosion reaction in TS steel of a banded ferrite/bainite/martensite structure. Obvious pitting and grain boundary corrosion were visible on both DS and TS ferrite/pearlite, with severe damage on ferrite and moderate damage on pearlite at 1h of corrosion test and more degradation between interfaces after t = 2 h. The pitting corrosion initiated as particular area of microstructure was formed from imperfection or weaken passive layer, as well as by atomic-scale impurity that accelerated corrosion reaction [20]. Moreover, grain boundary corrosion occurred since there was a discrepancy in density on each phase that contacted through an interface in which allowed O_2_ to diffuse on the boundaries [21]. For DS and TS ferrite/bainite phase, severe damage by mostly pit growth occurred on ferrite with slight deterioration on bainite at t = 1 h of corrosion, then pitting and boundary-to-boundary corrosion on both phases at t = 2 h. This was due to the ferritic, which had much higher Fe content than bainitic, thus, ferrite acted as cathode, whereas bainite-plate-like structure referred to anode [22]. With the compacted lath-shaped crystal grain of martensitic, almost no surface alteration occurred on this phase after 2 h of corrosion test, while pitting and boundary corrosion happened on ferrite since t = 1 h. This preferential attacked on ferrite, due to martensite phase transformed from body-centered cubic (BCC) into body-centered tetragonal (BCT) structure that offered least Fe content than other phases [23]. Similarly, ferrite defined as anode, while martensite classified as cathode. Thus, with the lowest Fe, the content possesses the highest corrosion resistance—martensite, then bainite, pearlite, and lastly ferrite phase. Although SEM images demonstrated a clear evolution of phase modification as the function of corrosion test times, further topographical investigation on its surface roughness is required to prove the preferential attack of certain phase phenomenon by 3DLSCM analysis.

**Figure 6:**
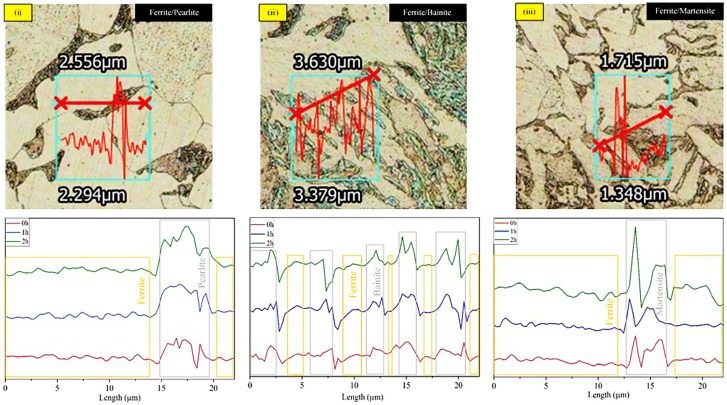
3DLSCM imaging of different corrosion behavior from each phase through line scan profile for (i) DS ferrite/pearlite, (ii) TS ferrite/bainite, and (iii) TS ferrite/martensite in immersion of 3.5 wt. % NaCl solution.

The 3DLSCM is capable of analyzing surface roughness of each microstructural interface through its combination of optical and laser imaging features. In Figure 6, it can be observed that different pairs of phases were examined on its roughness before and after corrosion, thus, offered line scan profile changes of each individual phase reacted to the corrosion attack. These pair of phases such as DS ferrite/pearlite, TS ferrite/bainite, and TS ferrite/martensite, were examined in such a way that overall corrosion behavior can be concluded from the mean surface roughness depth, Rz, in nano-scale unit quantitative data. The summary comparison of Rz values from interconnection between two phases before and after 2 h of corrosion test in DS and TS steels were represented in Table 2.

It can be seen that the lowest Rz value alteration was on the sample with the lowest quenching rate. This was due to degradation of both phases at different corrosion rates and initiation of grain boundary corrosion between interfaces. The corrosion was less affected on pearlitic than ferritic phase because it is transformed from a banded lamellar network of ferrite and cementite (Fe_3_C) [19,24]. On TS steels, the surface roughness difference was more than double the original surface, as more preferential attack on ferrite phase, since bainite created from groups of fine non-lamellar structures in Fe_3_C that offered platelet microstructure. This plate-like structure will enhance corrosion resistance properties more than pearlite, due to weak or unprotected alpha (α) phase in the lamellar of pearlitic phase, which promotes corrosion reaction [25]. The most substantial Rz value—nearly triple the initial surface of steel—occurred on the ferrite/martensite, since martensitic phase remained almost unmodified with severe damage on ferritic phase throughout corrosion process. This martensite has lath-shape with highly deformed structure, as it is established from diffusion less transformations that do not allow carbon atom to diffuse out of the crystalline structure, which possesses higher C content than other phases [23]. These steady protective lathe structures offer the least propensity toward corrosion attack. Nonetheless, the preferential corrosion attack on the highest Fe content phase in both DS and TS steels. As result, corrosion properties of each microstructural phase can be defined through its crystalline structure properties and significant roughness changes on Rz value, hence, the most preferential attack on crystalline phase—ferrite—and consecutively followed by pearlite, bainite, and martensite.

**Figure 7:**
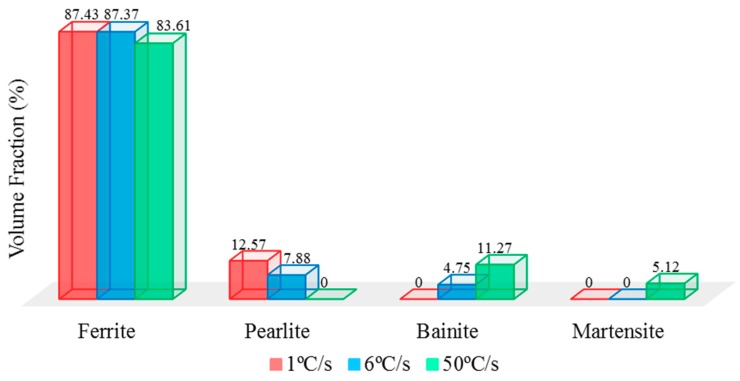
Comparison of volume fraction for generated phase as the effect of different quenching rate parameter on carbon steel samples.

Further investigation on volume fraction of each phase to confirm the presence of bainite and martensite phases in TS steels that improved the corrosion resistance properties than DS steels. Volume fraction of each phase was analyzed by image analyzer software, ImageJ. The images of four different areas on each sample was compiled to obtain the average volume fraction of each phase by grey-style mode, the comparison of volume fraction is indicated in Figure 7. The volume fraction of ferrite phase remained similar for all samples in which pearlite existed around 12.57% and 7.88% on sample-1 °C/s and sample-6 °C/s, respectively. The effect of decreased pearlite percentage had been attributed to higher overall corrosion resistance, especially on tertiary phases—bainite and/or martensite on higher-level quenching rate samples. Additionally, the absence of pearlite phase on sample-50 °C/s had confirmed its microstructural influence on increased susceptibility toward corrosion degradation. Although existence of bainite accounted to 11.27% and martensite contributed around 5.12% on sample-50 °C/s, its corrosion resistance properties on this grade of carbon steel had been substantially improved. As results, this analysis has clearly shown that different volume fractions of each phase will lead to dissimilarity on corrosion resistance properties and has fulfilled the agreement of microstructural analysis. This heat treatment has been designed in the way to have this effect, and to explore other parameter such as hardness properties.

After microstructural, electrochemical, and volume fraction measurements were investigated, the mechanical properties were tested to assess the enhancement in overall hardness, due to the imperative existence of the tertiary phase. From Table 3, the hardness value of sample with 1 °C/s quenching rate had 13.53% and 24.06% less than those with higher quenching rates at 6 °C/s and 50 °C/s. With the presence of DS, higher volume fractions in ferrite/pearlite phase led to decreases in hardness properties for 3.76% after 2 h of corrosion, while hardness value loss on TS steel at quenching rate 6 °C/s and 50 °C/s accounted to approximately 1.99% and 1.21%, respectively, at 2 h of corrosion test. Moreover, as the quenching rate was set to highest level, it led to the increase of martensite phases benefit to improve the hardness properties. The main reason of this occurrence was because of compacted body-centered tetragonal (BCT) structure that promoted shear deformations in which generated a tremendous group of dislocations—highly stressed structure [23,26]. Bainite is created from Fe_3_C and dislocation-rich ferrite that forms intermediate hardness between martensite and pearlite phases. Pearlite is built from mostly ferrite phase with low percentage of Fe_3_C that forms lamellar network and has relatively higher hardness properties than ferrite. However, the stress relief that included the recovery of dislocations will lead to the reduction in hardness properties at lower-level of quenching rate. In summary, the hardest microstructural phase is martensite, followed by bainite, pearlite, and ferrite. Therefore, with the presence of steady solid phases, bainite and martensite with balanced volume fraction of those phases not only will lead to enhancement in corrosion resistance, but also hardness properties in TS steels.

## 4. Conclusions

Electrochemical, microstructural, and mechanical properties have been performed to study the different microstructural engineering through heat treatment process that vary its corrosion resistance and hardness properties in low carbon steels. By performing in-situ observation on confocal microscopy, the secondary and tertiary phases had been achieved through heat treatment in the agreement of CCT diagram to generate DS and TS grades of steel. Through standard corrosion test using Tafel plot, the presence of bainite/martensite in TS steels has higher corrosion resistance up to 5.31% with topographical imaging by SEM analysis and 3DLSCM line scan profile on surface roughness of each phase, where we delivered a different perspective on enhancement of its corrosion resistance. Having this tertiary phase had proportionally improved the hardness properties up to 27.34% to precise Vickers hardness measurement. These findings have advanced prerequisites for new materials and various naval, marine, and automotive structural applications, e.g., longitudinal beams. The effect of C content on individual phase has led to different corrosion behavior in which higher contents of martensite had the best corrosion resistance than other phases. Overall corrosion in these DS and TS steels were pitting and grain boundary corrosion. The hardness was improved as more bainite/martensite occurred on TS steels, as they possessed lath-or plate-like shape that were robust, meaning that it also enhanced wear resistance. As abrasion, corrosion, and hardness properties of steel have always been sought for many industrial applications to reduce unnecessary costs corresponded with parts failure and/or short maintenance lifecycle, it is imperative to improve these properties for cost effectiveness. Therefore, it can be predicted that TS steels with higher percentage of bainite/martensite will have benefits in manufacturing applications, as this delivers enhancement in corrosion resistance, hardness, and integrity for various applications. As results, cost reduction can be achieved by heat treatment in different quenching process, thus, avoiding additional alloying elements.

## Figures and Tables

**Table 1:** Electrochemical quantitative data on different quenching rate of DS and TS steels in 3.5 wt. % NaCl solution.

Sample-ID	Potential (mV vs. SCE)	i_corr_ (mA/cm^2^)
1 °C/s—F + P	−712 ± 4	2.260 ± 0.002
6 °C/s—F + P + B	−691 ± 6	2.221 ± 0.005
50 °C/s—F + B + M	−633 ± 4	2.146 ± 0.007

**Table 2:** Comparison of the effect of corrosion at different time on mean surface roughness depth (Rz) and ΔRz values after 2 h of corrosion in nano-scale for each microstructural interface.

Sample-ID	Rz (nm)			ΔRz (nm) after 2 h
	0 h	1 h	2 h	
1 °C/s—F + P	262 ± 4	306 ± 5	430 ± 5	168
6 °C/s—F + P + B	251 ± 7	357 ± 2	579 ± 3	328
50 °C/s—F + B + M	367 ± 5	488 ± 4	913 ± 6	546

**Table 3:** Effect of different quenching rate on hardness properties before and after immersion into 3.5 wt. % NaCl solution, as per agreement with ASTM E140.

Sample-ID	Hardness (HV)			ΔHardness (HV) after 2 h
	0 h	1 h	2 h	
1 °C/s—F + P	133	130	128	5
6 °C/s—F + P + B	151	149	148	3
50 °C/s—F + B + M	165	164	163	2
